# Clinical and genetic characterisation of dystrophin-deficient muscular dystrophy in a family of Miniature Poodle dogs

**DOI:** 10.1371/journal.pone.0193372

**Published:** 2018-02-23

**Authors:** Lluís Sánchez, Elsa Beltrán, Alberta de Stefani, Ling T. Guo, Anita Shea, G. Diane Shelton, Luisa De Risio, Louise M. Burmeister

**Affiliations:** 1 Neurology/Neurosurgery Service, Centre for Small Animal Studies, Animal Health Trust, Kentford, Newmarket, Suffolk, United Kingdom; 2 Department of Pathology, School of Medicine, University of California San Diego, La Jolla, California, United States of America; 3 Kennel Club Genetics Centre, Animal Health Trust, Kentford, Newmarket, Suffolk, United Kingdom; Faculty of Animal Sciences and Food Engineering, University of São Paulo, BRAZIL

## Abstract

Four full-sibling intact male Miniature Poodles were evaluated at 4–19 months of age. One was clinically normal and three were affected. All affected dogs were reluctant to exercise and had generalised muscle atrophy, a stiff gait and a markedly elevated serum creatine kinase activity. Two affected dogs also showed poor development, learning difficulties and episodes of abnormal behaviour. In these two dogs, investigations into forebrain structural and metabolic diseases were unremarkable; electromyography demonstrated fibrillation potentials and complex repetitive discharges in the infraspinatus, supraspinatus and epaxial muscles. Histopathological, immunohistochemical and immunoblotting analyses of muscle biopsies were consistent with dystrophin-deficient muscular dystrophy. DNA samples were obtained from all four full-sibling male Poodles, a healthy female littermate and the dam, which was clinically normal. Whole genome sequencing of one affected dog revealed a >5 Mb deletion on the X chromosome, encompassing the entire *DMD* gene. The exact deletion breakpoints could not be experimentally ascertained, but we confirmed that this region was deleted in all affected males, but not in the unaffected dogs. Quantitative polymerase chain reaction confirmed all three affected males were hemizygous for the mutant X chromosome, while the wildtype chromosome was observed in the unaffected male littermate. The female littermate and the dam were both heterozygous for the mutant chromosome. Forty-four Miniature Poodles from the general population were screened for the mutation and were homozygous for the wildtype chromosome. The finding represents a naturally-occurring mutation causing dystrophin-deficient muscular dystrophy in the dog.

## Introduction

Duchenne muscular dystrophy (DMD, OMIM 310200) is an X-linked, recessive disorder of humans that affects approximately one in 3,500–5,000 newborn, live males [1, 2]. DMD typically demonstrates a strong familial link, although approximately one third of cases occur sporadically [3]. It is caused by mutations in the *DMD* gene, resulting in deficiency or absence of functional dystrophin [4, 5]. The clinically milder Becker Muscular Dystrophy (BMD, OMIM 300376) is also caused by mutations in the *DMD* gene.

*DMD* is one of the largest genes in the human genome, spanning approximately 2.5 Mb and encoding 79 exons [6]. Mutations which disrupt the reading frame so that virtually no protein is synthesised typically cause DMD, while those that maintain the reading frame and result in abnormal but partially functional dystrophin cause the milder BMD [7]. The *DMD* gene is highly conserved and homologues have been identified in both vertebrates and invertebrates, including the dog (*Canis familiaris*) [8].

Dystrophin is a large cytoskeletal protein predominantly present in skeletal and heart striated muscle [9]. It connects the muscle fibre cytoskeleton to the extracellular matrix, providing stability during contraction [10]. Dystrophin deficiency therefore results in progressive degeneration of these tissues [5]. DMD is characterised by progressive muscle weakness, respiratory insufficiency, cardiomyopathy and death in the late teens or twenties [11]. Although it is currently incurable, gene and stem cell therapies and dystrophin restoration approaches are under investigation [11–13].

Dystrophin deficiencies have also been described in animals, and the molecular basis has been identified in species such as rats, mice, cats, pigs and many breeds of dogs [14–27]. Dystrophin deficiency in the mdx mouse results in a milder phenotype compared to that of DMD [8]. However, the phenotype of dystrophin-deficient dogs has significant resemblance to human DMD, making affected dogs good animal models [28] and this has enabled the application of DMD canine models in preclinical gene therapy research [29].

In this report we describe dystrophinopathy, and identify and characterise the underlying mutation, in a family of parti–coloured Miniature Poodles (MPs).

## Materials and methods

All dogs clinically examined were pet dogs examined and treated at the request of their owners–they were not part of a research colony. All clinical and diagnostic veterinary procedures on animals in this study were performed with informed owner consent by licensed veterinary surgeons. All samples were obtained from privately owned pet dogs with the owners’ consent. The majority of DNA samples were obtained using a non-invasive buccal swab. Where DNA was obtained from blood, this sample was residual to blood drawn for diagnostic veterinary purposes, and not specifically for the purposes of research. Additionally, no *in vivo* experiments were undertaken. All clinical examinations and diagnostic investigations were conducted during the course of veterinary care and not specifically for research purposes. The study was approved by the Animal Health Trust Ethics Committee.

### Clinical evaluation

**Fig 1:**
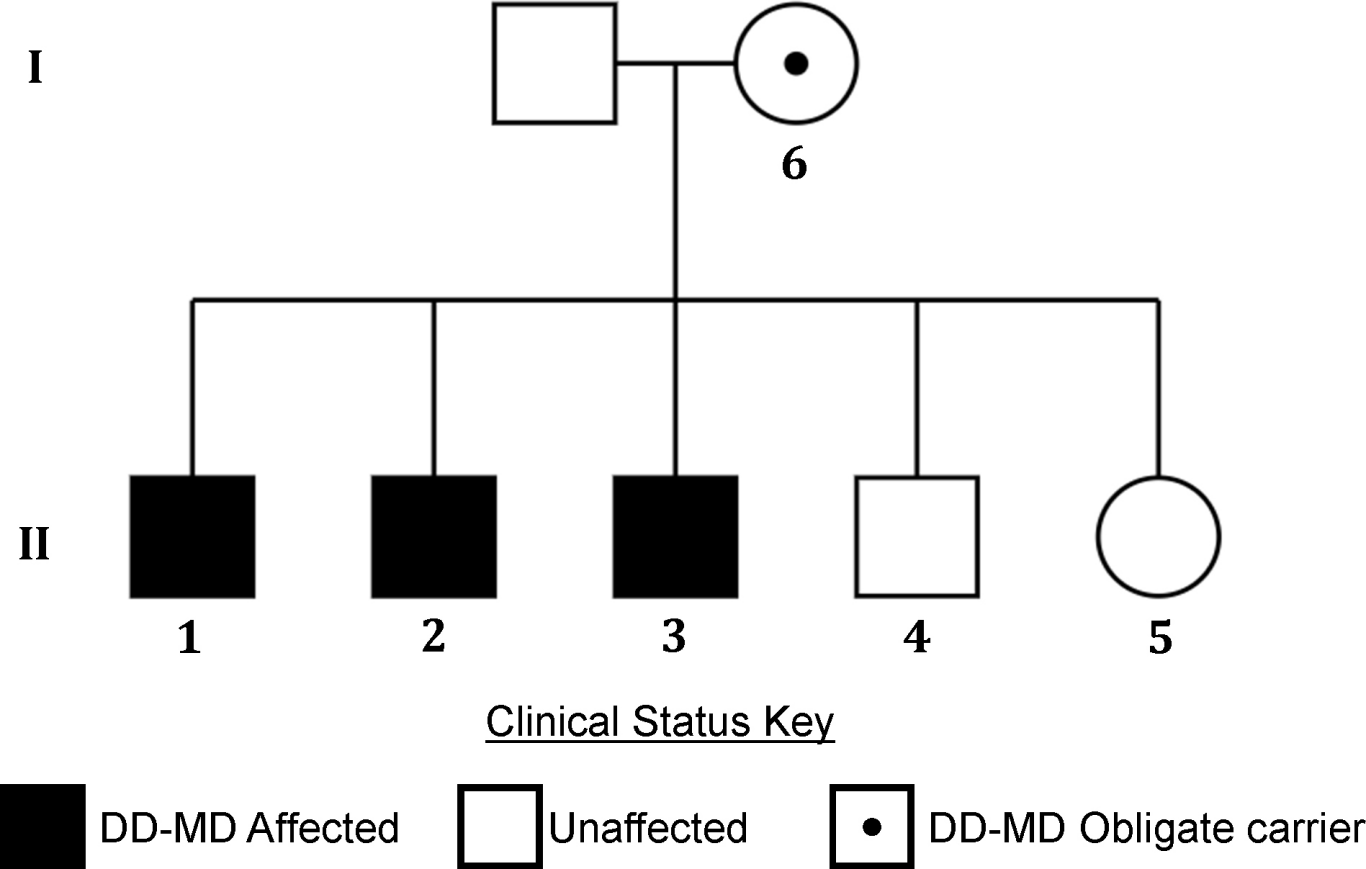
Pedigree showing the dystrophin-deficient muscular dystrophy affected MP family. The litter is made up of three affected males, one unaffected male and one unaffected female. Both parents are also unaffected. DNA samples were available from dogs 1–6.

Four full-sibling, intact male, client-owned MPs from the United Kingdom (dogs 1–4, Fig 1) were evaluated at the Neurology/Neurosurgery Department of the Animal Health Trust. These dogs were Harlequin-coloured, a subtype of Parti coat colouring. The litter consisted of four males and one female. The female puppy and the dam were reported to be clinically normal; no information was available regarding the clinical status of other relatives. Standard clinical and neurological examinations were performed in dogs 1–4. Magnetic resonance imaging (MRI), electromyography and muscle biopsies were conducted in dogs 1 and 2 under general anaesthesia. MRI of the brain (dogs 1 and 2) and neck (dog 1) was performed with a 1.5T scanner (Signa EchoSpeed MRI, GE Healthcare, Milwaukee, Wisconsin, USA). Transverse, dorsal and sagittal T2-weighted (3,000, 4,000/83-86; TR/TE) (T2W) sequences of the brain were obtained in both dogs. Transverse fluid attenuation inversion recovery (8002/98), transverse T1-weighted (600/12) (T1W) and transverse (dogs 1 and 2) and dorsal (dog 1) T1W sequences after intravenous gadolinium (0.1 mmol/kg of gadopentetate dimeglubine) (460–600; 12–13) were also obtained. Transverse T2* gradient echo (500; 15) sequence was acquired for dog 2. The MRI of the neck for dog 2 consisted of dorsal and sagittal plane T2W sequences (3000; 86). Electromyography included assessment of tongue and temporalis muscles, epaxial muscles, and pelvic and thoracic appendicular muscles (including flexors and extensors at various depths) using digital electrodiagnostic equipment (Medelec Synergy, Oxford Instruments).

### Sample collection

Open muscle biopsy samples were obtained from the right infraspinatus, supraspinatus and epaxial muscles in dog 1 (aged four months) and from the brachial triceps, infraspinatus and cranial tibial muscles in dog 2 (aged nine months).

Residual blood from dog 2, originally taken as part of a veterinary diagnostic procedure and collected into an EDTA tube, was used for genetic investigations. Buccal swab samples were also collected from all members of the litter and the mother, as well as from 44 MPs unrelated to this MP family (including 19 parti-coloured).

### Histopathology, immunohistochemistry and western blotting

Following collection, biopsy specimens were either flash-frozen in isopentane precooled in liquid nitrogen and stored at -80°C until further evaluation, or immersion-fixed in 10% neutral buffered formalin and embedded in paraffin. A standard panel of histochemical stains and reactions was performed on 8 μm muscle cryosections [30].

Additional cryosections were used for immunofluorescent stainings. Antibodies included mouse monoclonal antibodies against the rod domain (1:100, NCL-DYS1, Leica Biosystems) and carboxy terminus (1:100, NCL-DYS2 Leica Biosystems) of dystrophin, α-sarcoglycan (1B4, 1:200, gift from Professor Eva Engvall), β-sarcoglycan (1:100, NCL-βSG, Leica Biosystems) and γ-sarcoglycan (1:100, NCL-γSG, Leica Biosystems), utrophin (1:20, NCL-DRP2, Leica Biosystems), spectrin (1:10, NCL-SPEC2, Leica Biosystems) and developmental myosin heavy chain (1:20, F1.652 Developmental Studies Hybridoma Bank), and a rabbit polyclonal antibody against laminin α2 (1:200, gift from Professor Eva Engvall). Stainings were visualised using immunofluorescent techniques as previously published [31]. Fluorescent labels included FITC-conjugated goat anti-mouse IgG (Jackson Immuno Research 111-095-003) and FITC-conjugated goat anti-rabbit IgG (Jackson Immuno Research 115-095-003). Representative areas were chosen by visual inspection of the entire section. Archived control canine limb muscle was similarly processed.

For western blotting, muscle proteins were extracted using RIPA buffer. Samples were centrifuged at 9,000 g for 10 minutes to remove insoluble debris. Soluble proteins were resolved by 4–12% Nu-PAGE Bis-Tris gels (Invitrogen) and transferred to nitrocellulose membranes (Hybond; Amersham Biosciences). All membranes were stained with Ponceau (Sigma-Aldrich) to evaluate the amount of loaded proteins. Membranes were blocked for one hour in Tris-buffered saline Tween (TBS) containing 5% powdered skim milk and incubated overnight with the following primary antibodies: anti-dystrophin NCL-DYS1 and NCL-DYS2 (1:1000, both from Novocastra) and β-actin (1:2000 from Sigma) for loading control. Horseradish peroxidase (HRP)-conjugated secondary antibody (1:30,000, Jackson ImmunoResearch and Termo Scientific) was used to detect antibodies with SuperSignal chemiluminescence kit (ECL Thermo Scientific).

### Genetic investigation

Genomic DNA was extracted from buccal swabs using a QIAamp DNA Blood Midi Kit (Qiagen) and from blood using the Nucleon BACC2 DNA extraction kit (GE Healthcare Life Sciences) using the manufacturers’ instructions. Whole genome sequencing (WGS) of a single affected dog (dog 2) was outsourced to Edinburgh Genomics, University of Edinburgh. Paired end Illumina sequencing of a TruSeq Nano DNA library was undertaken, with a read length of 150 bp and resulting in ~30x coverage of the genome. Reads were aligned to the CanFam3.1 canine genome build using Burrows-Wheeler Aligner (BWA) [32] and variants called using Genome Analysis Toolkit (GTAK) [33]. WGS aligned with CanFam3.1 was visualised using Integrative Genomics Viewer (IGV) and compared with WGS from 113 non-MP dogs without signs of muscular dystrophy. The MP genome sequencing dataset can be accessed via the European Nucleotide Archive (ENA accession number: PRJEB22955).

Primers for DNA amplification were designed with Primer3 (S1 Table) [34]. PCRs were carried out in 12 μL volumes consisting of 0.2 mM dNTPs (NEB), 0.83 μM of each primer, 1x PCR buffer, 0.1 U of Qiagen HotStarTaq Plus DNA polymerase, where necessary 1x Q solution (Qiagen), and template DNA. Thermal cycling conditions were used according to the manufacturer’s instructions and melting temperatures, elongation times and the use of Q solution additive are summarised in S1 Table.

PrimeTime probe-based assays for the quantitative PCR (qPCR)-based genotyping were designed using the PrimerQuest® Design tool (Integrated DNA Technologies). Assays contained ZEN™/Iowa Black FQ Double-Quenched Probes and were targeted to regions of the X chromosome with unique sequences and without structural variations (assessed using the Ensembl genome browser). The target assay (DMD_del) was designed to target within the DMD gene (Primer1: GCTGCTTCCCAAACTGAAATATG; Primer2: CCCTTGGCAGATTAAGGGTTAG; Probe: 6-FAM- ACAAAGCACTCTGCCAGTGATCCA) and the reference assay (DMD_ref) to target elsewhere on the X chromosome (X:123,728,608–123,728,746; Primer 1: ACTCACATACTCACATGGCAAG; Primer 2: TCCTGTCAGTTTATCTACCAAGAAC; Probe: HEX-TTTCACCCTCTACCCATGCAGAGC). Quantitative PCR was carried out on an Illumina Eco qPCR machine in 6 μL reactions, comprising 1x KAPA Probe Fast qPCR Master Mix, 1x DMD-del, 1x DMD-ref, 1x ROX and 1 μL DNA template. Thermal cycling comprised 50°C for 2 minutes, 95°C for 5 minutes, followed by 45 cycles of 95°C for 10 seconds and 61°C for 30 seconds. Reaction efficiencies were calculated using a five-point 5x serial dilution to create a standard curve. DMD_del and DMD_ref reaction efficiencies were estimated at 102.0% and 98.5% respectively, with R^2^ values both >0.995. Reactions were performed in triplicate, CT values of DMD_del were normalised to those of DMD_ref, and comparisons between the unknowns and a non-MP male reference sample were performed with the Δ ΔCT method [35].

## Results

### Clinical investigation

The pedigree of the dystrophin-deficient muscular dystrophy (DD-MD) affected MP family is illustrated in Fig 1. The clinical and diagnostic findings, treatment and outcome of the four full-sibling, intact male, client-owned MPs from the United Kingdom (dogs 1–4) are summarised in S2 Table. The four dogs (dogs 1–4) were presented at the ages of four to 19 months. Dog 4 was clinically normal. Dogs 1–3 had a history of reluctance to exercise, progressive and generalised muscle atrophy and a stiff gait affecting all four limbs from eight weeks (dogs 1 and 2) and six months (dog 3) of age. Dogs 1 and 2 also presented with lethargy, hyporexia, slow and poor physical development, learning difficulties and episodes of abnormal behaviour. During these episodes dogs 1 and 2 started crying and biting for a few seconds, without a specific trigger, after which they were extremely unsettled for 2–3 minutes. The owner of dog 2 also reported episodes of decreased awareness, independent of the aforementioned crying and biting episodes. Pre-referral haematology and serum biochemistry profiles of dogs 1–3 revealed a serum creatine kinase (CK) activity between 8,000 and 96,523 IU/L (reference interval 21 to 56 IU/L). The haematology and serum biochemistry panels of dog 4 were unremarkable. Serological titres for *Toxoplasma gondii* and *Neospora caninum* in dogs 1 and 2 were negative.

Physical and neurological examinations of dogs 1–3 revealed a poor body condition and generalised muscle atrophy. Neurological examination of dogs 1 and 2 also revealed reluctance to exercise, an abnormal posture characterised by extension of the limbs and lumbar kyphosis, a stiff gait affecting all four limbs and slightly delayed postural reactions in the pelvic limbs. Dog 1 exhibited cranial and cervical hyperaesthesia. No discomfort was elicited on palpation of the muscles, spine or head in the other affected dogs. Physical and neurological examinations of dog 4 did not reveal any abnormalities.

The neurolocalisation for dogs 1–3 was to the neuromuscular system. Due to the history of abnormal behaviour episodes, concurrent forebrain involvement was also considered in dogs 1 and 2. Given the hyperaesthesia, cervical involvement in dog 1 was not excluded. Differential diagnoses for the muscular lesions included degenerative myopathy (muscular dystrophy or other congenital myopathies), polymyositis (inflammatory/infectious/immune-mediated), metabolic myopathy and nutritional myodegeneration. Differential diagnoses for the potential forebrain lesions included developmental anomalies, degenerative encephalopathies and meningoencephalitis (infectious/immune-mediated). The cervical hyperaesthesia detected in dog 1 may have been a consequence of the presumptive myopathy or the potential intracranial disease.

The owners of the unaffected dog (dog 4) and dog 3 only consented to measurement of the serum CK level, which was unremarkable and 47,185 IU/L respectively. Repeated serum CK concentrations in dogs 1 and 2 were 157,232 and 225,674 IU/L respectively. Quantification of amino and organic acids, carbohydrates and mucopolysaccharides in the urine of dog 1 was unremarkable. Echocardiography of dog 2 did not reveal any significant abnormalities. Dog 1 did not undergo cardiac ultrasound. MRI of the brain (dogs 1 and 2) and neck (dog 1) were normal. Routine cerebrospinal fluid (CSF) analysis of dogs 1 and 2 was unremarkable and CSF real-time PCR for *T*. *gondii*, *N*. *caninum* and canine distemper virus were negative. Electromyography of dogs 1 and 2 revealed spontaneous fibrillation potentials and complex repetitive discharges in the infraspinatus, supraspinatus and epaxial muscles between the T2 and T4 vertebrae. All other muscles were normal on electromyography. Motor nerve conduction velocity of the ulnar nerve was within normal limits.

### Histopathology, immunohistochemistry and western blotting

**Fig 2:**
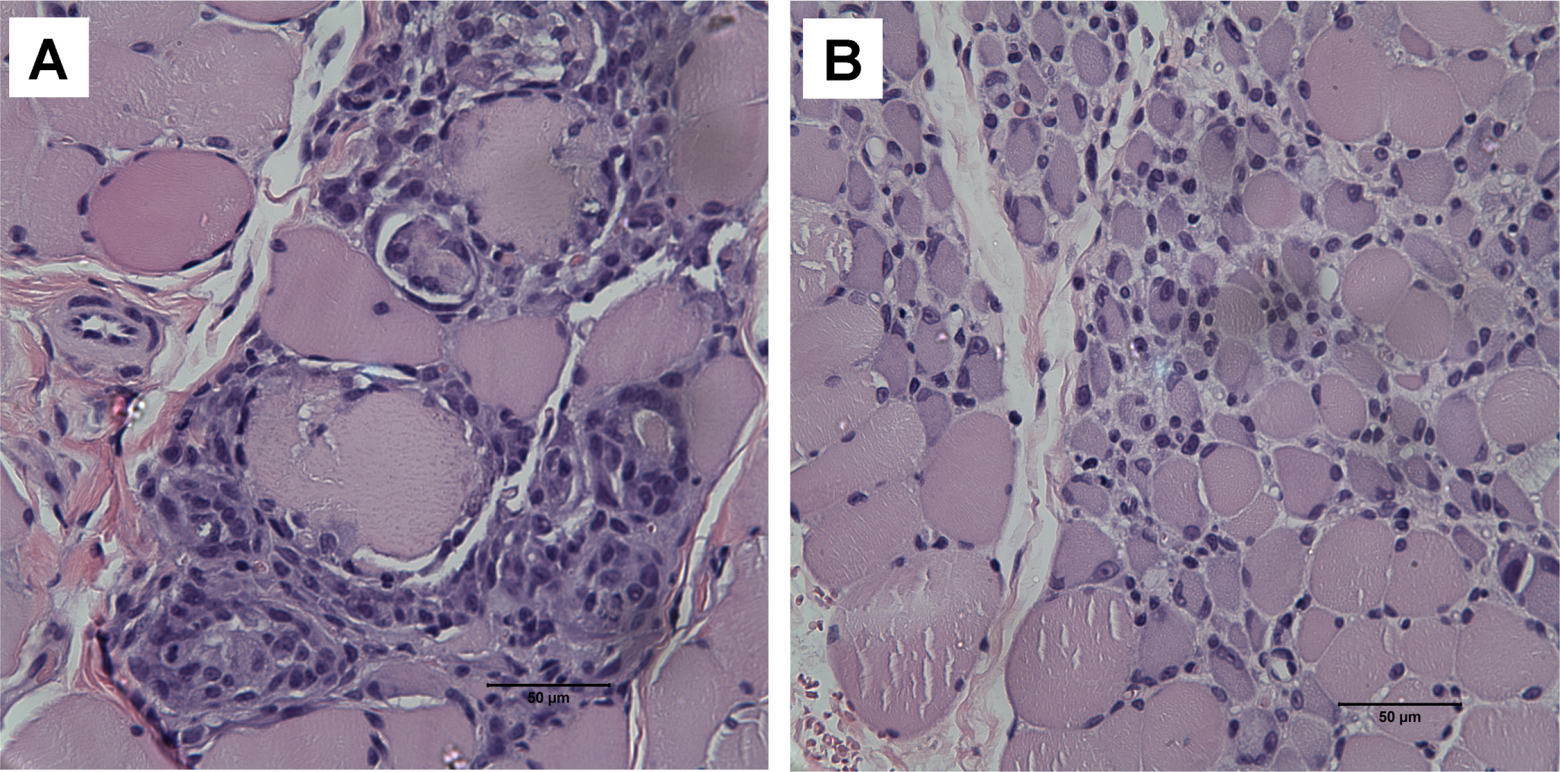
Representative histopathology of muscle cryosections from the infraspinatus muscle of dog 1. Multifocal areas of myofibre degeneration are characterised by scattered and groups of necrotic fibres undergoing phagocytosis (a) and large clusters of basophilic regenerating fibres (b) consistent with a dystrophic phenotype (H&E stain).

**Fig 3:**
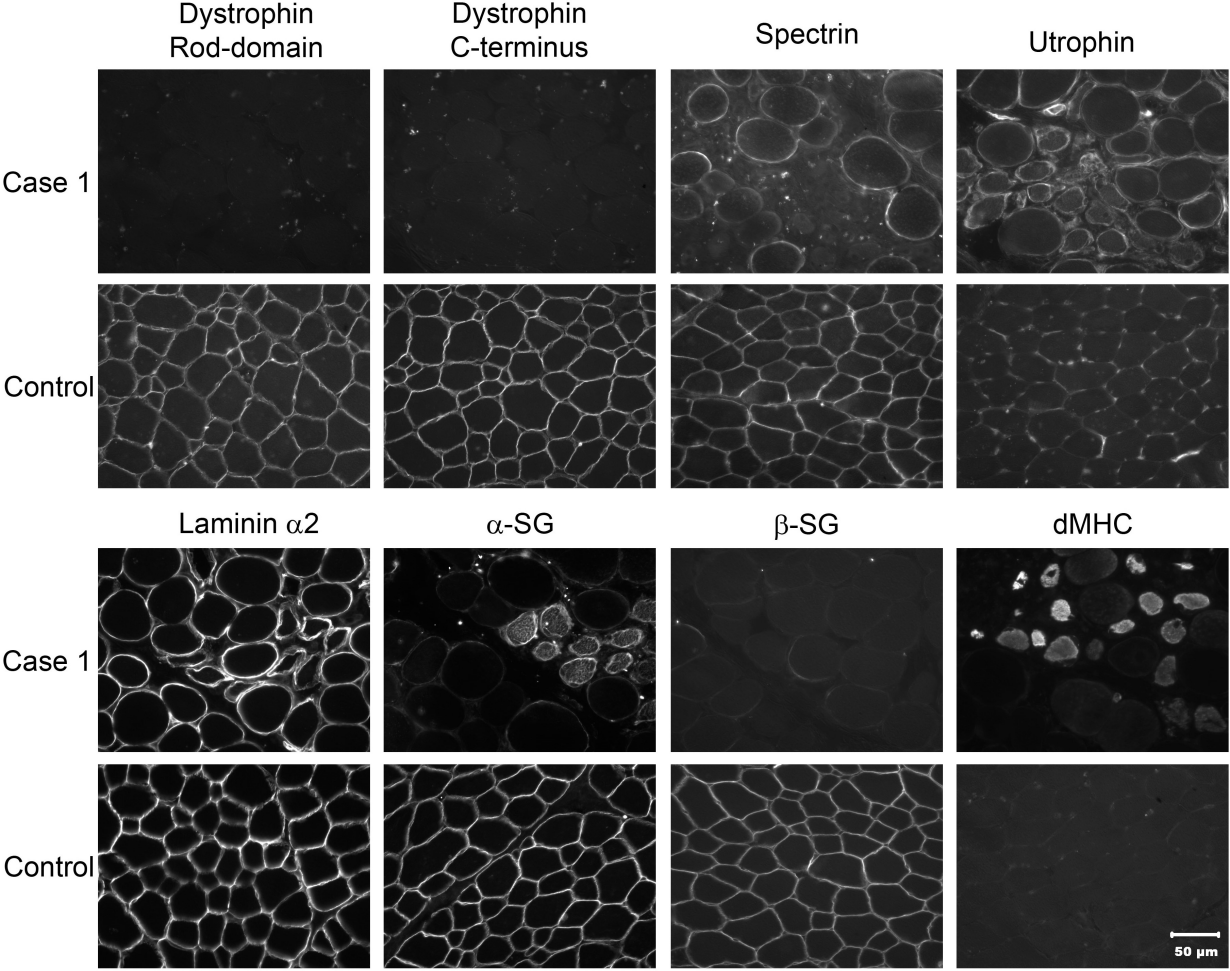
Immunofluorescent analysis of muscle biopsies for localisation of dystrophy associated proteins. Immunofluorescent staining of cryosections from dog 1 (dystrophic) and a control dog using antibodies to laminin α2, the rod domain and carboxy terminus (C-terminus) of dystrophin, dysferlin, α- and β-sarcoglycans (αSG and βSG respectively) and utrophin (DRP2). An antibody against spectrin was used as a control for membrane integrity. Staining was absent for the rod domain and carboxy terminus of dystrophin, decreased for β-sarcoglycans and similar for laminin α2, dysferlin, α-sarcoglycans and spectrin. Utrophin was visualized along the sarcolemma in the dystrophic dog but not on the sarcolemma of the control muscle. Regeneration was robust as shown by the antibody against developmental myosin heavy chain (dMHC).

**Fig 4:**
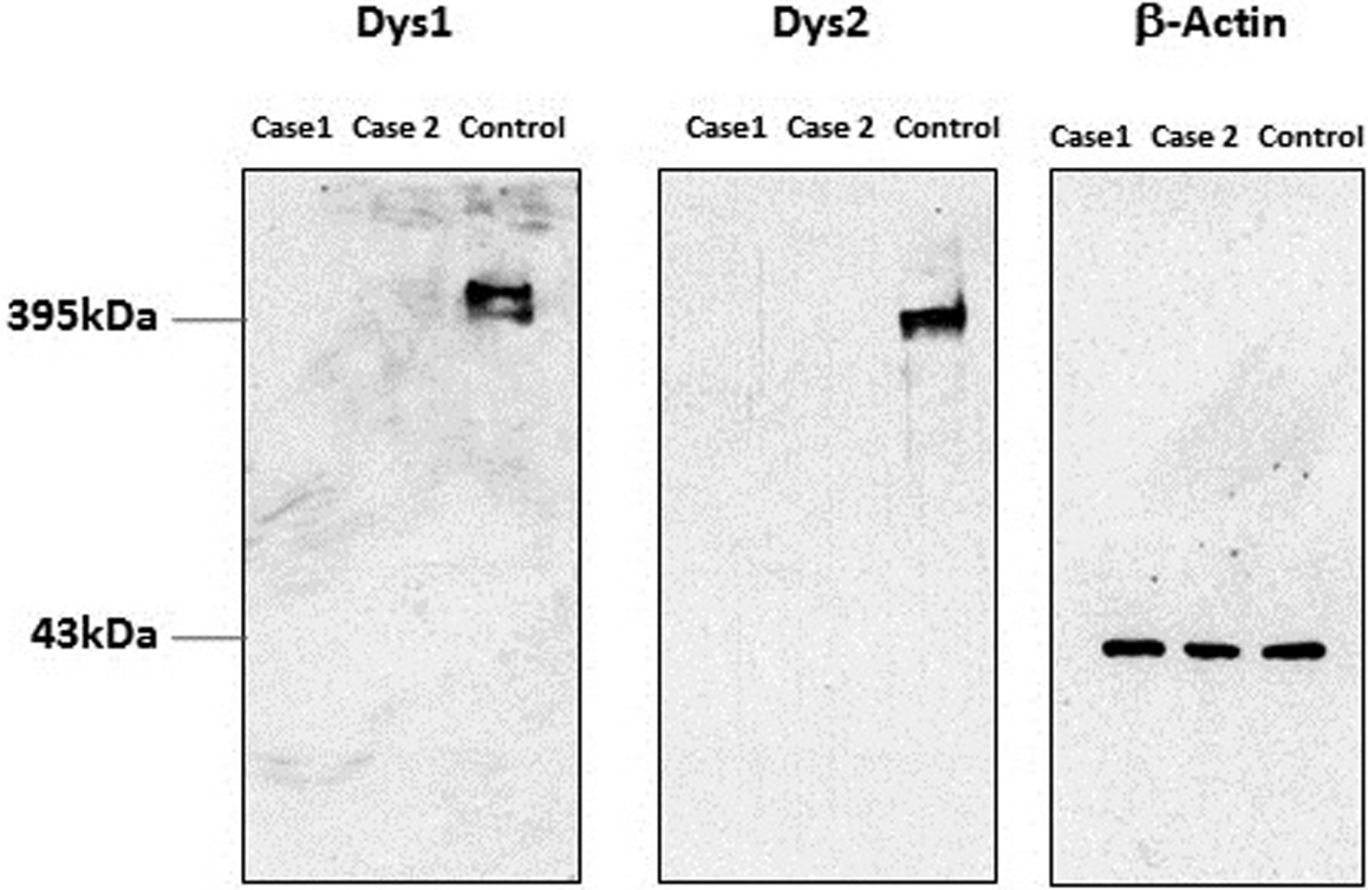
Western blotting of skeletal muscle from dystrophic dogs (dogs 1 and 2). Shown are stainings for the rod (DYS1) and carboxy terminus (DYS2) of dystrophin in the affected and control dogs. Bands are visible at approximately 395 kDA for the control muscle, but are not detectable in the dystrophic dogs. β-actin was used as a loading control and bands staining at a molecular weight of approximately 43 kDa confirm similar amounts of loaded protein in dystrophic and control dogs.

Biopsies of the right infraspinatus, supraspinatus and epaxial muscles were obtained from dog 1 and from the triceps, infraspinatus and cranial tibial muscles of dog 2. Histopathological examination of all muscle biopsies revealed a dystrophic phenotype characterised by numerous scattered and groups of necrotic fibres undergoing phagocytosis (a representative figure shown in Fig 2A) and large groups of basophilic fibres indicating regeneration (a representative figure shown in Fig 2B). Rare calcific deposits were observed in dog 2 (not shown). Compared to control archived canine limb muscle, immunofluorescent staining for both the rod domain and carboxy terminus (C-terminus) of dystrophin was absent, decreased for β-sarcoglycan and appropriate for laminin α2, dysferlin, α-sarcoglycans and spectrin (Fig 3). Staining for utrophin was observed along the sarcolemma of the dystrophic dog muscle but not in the control muscle. Regeneration was robust (Fig 3). Histopathology and immunofluorescent stainings were consistent with muscular dystrophy resulting from dystrophin deficiency. Western blotting confirmed the absence of both the rod domain and C-terminus of dystrophin obtained by immunostaining (Fig 4).

### Treatment and outcome

Dog 1 was treated with 0.2 mg/kg diazepam orally every eight hours, 2.0 mg/kg carprofen orally every 12 hours and 2–4 mg/kg tramadol orally every six to 12 hours as required. This treatment (aimed at improving the dog’s abnormal posture, stiff gait and cranial and cervical hyperaesthesia), continued until the dog was euthanised at 19 months of age by the referring veterinary surgeon due to suspected acute liver failure. The referring veterinary surgeon based their presumptive diagnosis on clinical signs (weakness, severe depression and anorexia), blood analyses (an increased serum alanine transaminase level– 800 IU/L, reference interval 20 to 155 IU/L) and abdominal radiographs (enlarged and irregular appearance of the liver). Post-mortem examination was not performed to confirm this presumptive diagnosis and we therefore cannot exclude the possibility that the treatment contributed to the suspected hepatopathy. Both diazepam and carprofen may have led to the increased serum ALT concentration.

Twenty-four hours after referral, dog 2 fractured the right radius and ulna due to a fall. Radiographs showed no abnormalities in bone density. The fracture was surgically repaired and the dog was intermittently treated with 0.1 mg/kg meloxicam orally every 24 hours and 0.4–0.8 mg/kg diazepam orally every eight hours (aimed at improving the dog’s abnormal posture and stiff gait), until euthanasia six years later, after fracturing the jaw while playing with another dog. Exercise intolerance and generalised muscle atrophy had progressed to the extent of difficulty swallowing. Post-mortem examination was not conducted in dog 2.

Dog 3 was lost to follow-up three months after the consultation, at 22 months old. At that stage the dog still retained reasonable quality of life.

### Molecular analysis

**Fig 5:**
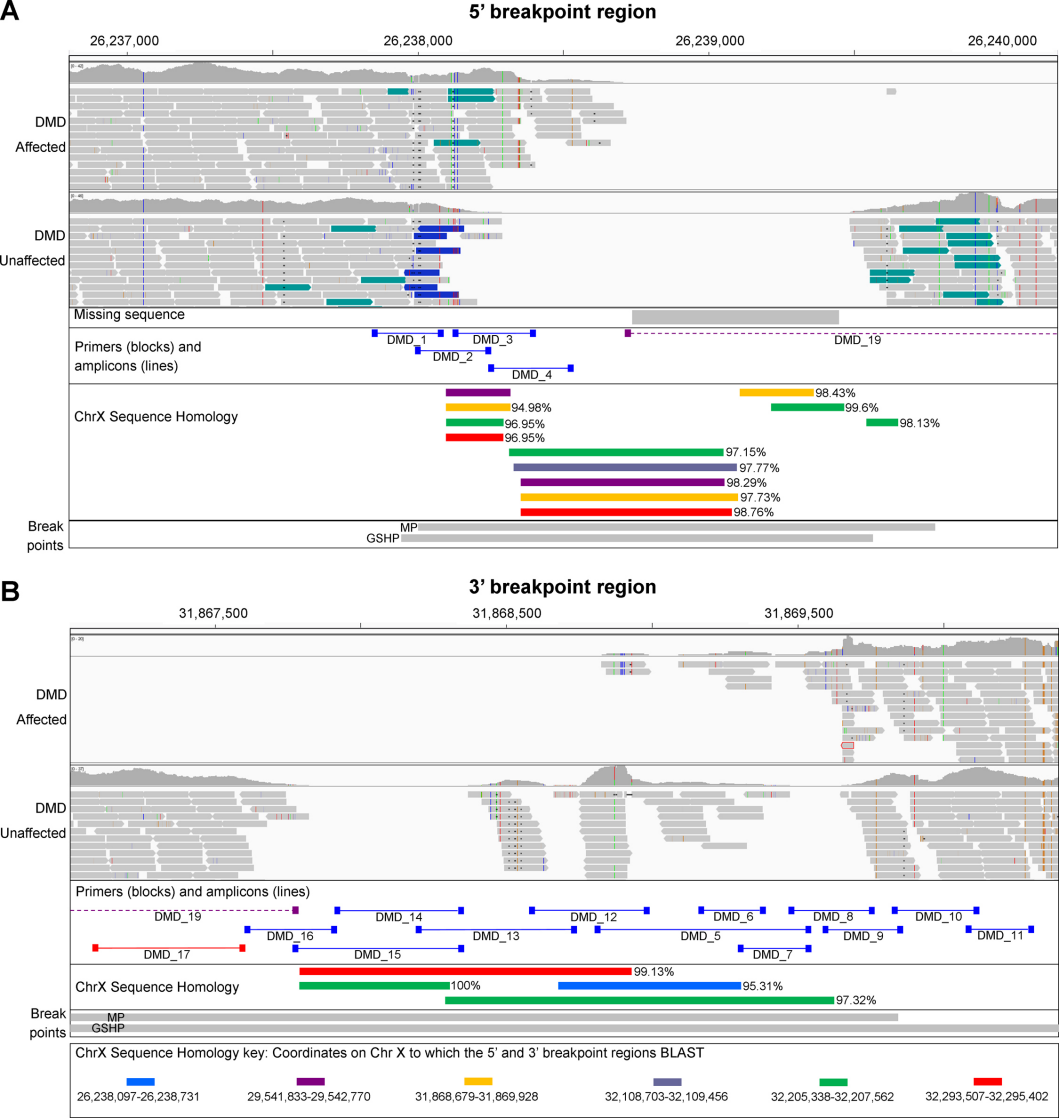
Breakpoint regions of the X chromosome deletion. IGV display of the 5’ (A) and the 3’ breakpoint regions (B), in a DMD-affected and a control dog. The complete absence of reads in the DMD-affected dog is suggestive of a >5.6 Mb deletion (approximate coordinates X:26,238,000–31,869,800). Part of the reference sequence, CanFam3.1, is unknown (missing sequence in (A)). The specific regions targeted for amplification with primers are indicated, as are the highly homologous regions and the regions containing the deletion breakpoints (for the MP and GSHP, as described by VanBelzen et al.).

The whole genome of dog 2 was sequenced to an average depth of 30x using paired end Illumina next generation sequencing. Due to the clinical evidence of dystrophin deficiency, genetic investigations were focused on the region of the *DMD* gene (CFAX: 26,290,714–28,333,431). Sequence reads, aligned to the canine reference sequence (CanFam3.1), were visualised using IGV. A region of >5.6 Mb on the X chromosome was devoid of reads, suggestive of a large genomic deletion (approximate coordinates: X:26,238,000–31,869,800) (Fig 5). Interestingly, this deletion is very similar to the 5.6 Mb deletion described in the German Shorthaired Pointer (GSHP) [36]. This region encompasses a number of genes, including the entire dystrophin gene. Comparison of WGS from our MPs and 113 non-MP controls revealed the complete absence of sequence reads in this region only in the MP. We were unable to define the precise breakpoints visually in IGV. In addition, part of the CanFam3.1 reference sequence is unknown (711 nucleotides from CFAX:26,238,732–26,239,442) missing sequencing in Fig 5A), and it is possible that the 5’ breakpoint is within this region. VanBelzen et al. were able to sequence this region from the relevant BAC clone and determined it was made up of 650 nucleotides [36].

In an attempt to define the deletion breakpoints experimentally we designed multiple pairs of primers to amplify overlapping fragments at the 5’ and 3’ breakpoint regions (DMD_1–18), and to amplify over the deletion breakpoints (DMD_19 and combinations of primers from DMD_1–18, Fig 5). Primers and expected and observed products are summarised in S1 Table. PCR amplification with DMD_1–3 was unsuccessful despite trying a variety of taq polymerases, additives and thermal cycling conditions. Amplification was successful for many of the flanking amplicons (DMD_4–18), although products of similar sizes were observed between the affected and control DNA for most primers (DMD_4–16, blue amplicons in Fig 5). Further analysis of the reference sequence revealed that much of the sequence targeted with these primers, including the missing 650 bp sequence, is highly homologous to regions elsewhere on the X chromosome (Fig 5). It is therefore possible, if not likely, that the PCR products observed are not specific to the breakpoint regions targeted. Indeed, the UCSC In-Silico PCR tool (www.genome.ucsc.edu/cgi-bin/hgPcr) confirmed that many of these primer pairs will likely amplify more than one region of the canine genome (S1 Table). With DMD_17 and DMD_18 primers, products were observed in the control, but not in the affected DNA (red amplicons in Fig 5), confirming the deletion breakpoints are downstream and upstream of these primer binding sites respectively. Finally, PCR amplification over the breakpoint could not be achieved with DMD_19 primers (purple amplicon in Fig 5) or with combinations of all primers, despite trying a variety of taq polymerases, additives and thermal cycling conditions. From PCR amplification and IGV we localised the 5’ and 3’ deletion breakpoints to 26,238,000–26,239,786 bp and 31,867,079–31,869,800 bp respectively (Fig 5).

To confirm that the region deleted in dog 2 is also deleted in the other affected males, but not in the three unaffected dogs, 11 primer pairs were designed to amplify DNA at approximately 500 kb intervals within the deleted region (S1 Table). PCR products were generated in the three unaffected dogs but not in the three affected dogs.

**Fig 6:**
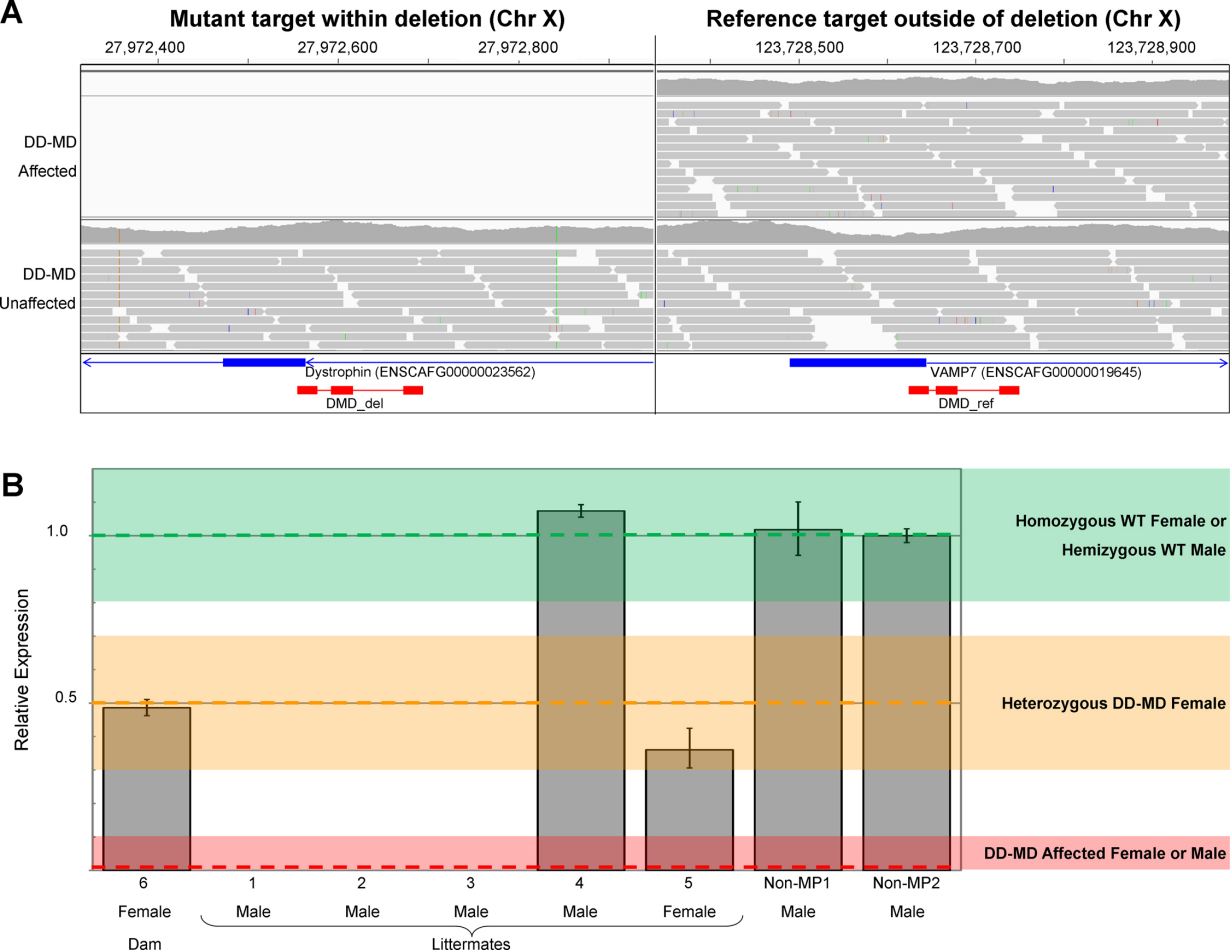
Quantitative PCR to genotype for the deletion. A) IGV display of sequence reads for the DD-MD affected MPs and unaffected control at the qPCR loci. The DMD-del assay targets part of the dystrophin gene within the deletion, and the DMD-ref assay targets elsewhere on the X-chromosome. CanFam3.1 genes are represented by blue bars, and qPCR primers and probes are represented by red bars. B) Levels of DMD_del genomic DNA, normalised against DMD-ref, relative to a non-MP male dog (non-MP2). DMD_del is completely absent from the DD-MD cases, is present at levels of approximately 0.5x in dogs 5 and 6, and at level of approximately 1x in dog 4 and the non-MPs. Error bars represent standard deviation.

As the deletion breakpoints remain unknown and we were unable to amplify over the breakpoints, we were unable to design a genotyping assay to target the deletion specifically. We therefore designed a relative qPCR-type assay to detect the copy number of the wildtype allele: the DMD_del assay targets the dystrophin gene within the deletion and the DMD_ref assay targets a unique region elsewhere on the X chromosome (Fig 6A). The qPCR assay was validated using the affected littermates (dogs 1–3), the unaffected male littermate (dog 4), the unaffected female littermate (dog 5) and the unaffected dam (dog 6) (Fig 1 and Fig 6B). DMD_del was completely absent from dogs 1–3, indicating they were hemizygous for the mutant allele. DMD_del was present at levels of approximately 0.5x in dogs 5 and 6 (both females), indicating they were heterozygous for the mutant allele. Finally, DMD_del was present as levels of approximately 1x in dog 4 and the non-MP controls, indicating males were hemizygous and females homozygous indicative of males hemizygous and females homozygous for the wildtype allele (Fig 6B). The mutation segregates with the DD-MD phenotype and is consistent with an X-linked recessive mode of inheritance.

To confirm that the mutation is not common in MPs, we screened 44 additional individuals, comprising 19 parti coloured and 25 solid coloured dogs. None of the individuals were hemizygous or heterozygous for the mutation, suggesting it is limited to this family of MPs and is most likely not present in the wider MP population.

## Discussion

In this study we sought to describe dystrophinopathy in a family of parti–coloured MPs, specifically the Harlequin coloured subtype, and to identify and characterise the underlying mutation.

Canine dystrophin deficiency is an X-linked recessive trait transmitted by a female carrier [37]. While it predominantly affects young purebred male dogs, affected female dogs have also been reported, although the clinical signs are less severe [9]. Similarly, in humans, most female *DMD* carriers are asymptomatic, but up to 20% exhibit mild to moderate muscle weakness [38] and 8% have dilated cardiomyopathy [39].

Dystrophin is predominantly present in skeletal and heart striated muscle [9] and connects the muscle fibre cytoskeleton to the extracellular matrix, providing stability during contraction [10]. Affected dogs present with clinical signs (which vary with breed) at a few weeks of age [40]. Typically the disease is characterised by generalised weakness associated with progressive muscle atrophy [41]; however, some muscles may be hypertrophied, such as the semitendinosus, semimembranosus and tongue muscles [41] or the cranial sartorius muscle in the Golden retriever [42]. Generalised hypertrophy has been described in a Rat Terrier dog [43] and Weimaraner [44]. Other reported clinical signs include exercise intolerance [41], plantigradism [28], dysphagia, dyspnoea, trismus [14], sialorrhoea [28], a short-stepping, rigid gait [40] and muscle and joint contractures [28]. Abnormalities in cardiac musculature have also been reported and may lead to heart failure [41].

Weakened skeletal muscles, amongst other factors, may lead to osteoporosis and traumatic fractures in DMD patients [45]. To the author’s knowledge, to date, no pathological fractures had been associated with DD-MD in dogs. We report here that dog 2 experienced multiple fractures, but X-rays revealed a lack of bone density abnormalities. However, it has been reported that 30–50% bone loss is required for abnormalities in bone density to be apparent on X-ray images [46]. Therefore, we cannot exclude the possibility that dog 2 may have had osteoporosis and the fractures reported could have been secondarily developed traumatic fractures. Unfortunately no post-mortem examination was performed to confirm the presence of osteoporosis.

Interestingly, despite their complete lack of dystrophin expression, the MPs presented here did not exhibit all the clinical signs that have been reported in canine DD-MD. Dogs 1 and 2 developed clinical signs at 8 weeks of age, whilst dog 3 developed clinical signs at 6 months of age. In addition, dogs 1 and 2 presented with more clinical signs and greater serum CK activities than dog 3. One possible reason for this is the fact that DD-MD is a progressive disease and clinical signs do not all appear simultaneously; some of the signs may have appeared at later stages of disease progression. Of all canine DMD models, only the GSHP also displays a deletion mutation encompassing the entire *DMD* gene [36]. Indeed, the deletion described in the GSHP is very similar in size and chromosomal location to that reported here for the MP [36] and we would expect to observe similar clinical signs as a result. Both the GSHP and MP models were presented with mainly similar clinical signs; however, the former were presented with trismus and (secondary) episodes of aspiration pneumonia, which the MP model did not display [47]. One of the GSHPs did not have echocardiographic signs of Duchenne’s cardiomyopathy at nine months of age, but had developed it by 21 months of age [47]. It was unknown whether the MP 2 presented here, which had no signs of cardiomyopathy on echocardiography when he was 9 months old, developed cardiomyopathy at a later stage. Similarly, both GSHPs developed hypertrophy of the cervical musculature and lateral head of the triceps brachii muscle at 43 months of age [47]. This was not observed in the MPs presented here at much younger ages. The phenotypic variation within MPs and between MPs and GSHPs may also reflect increased expression of other homologous proteins such as utrophin [28], the influence of extrinsic factors (e.g. nutrition, exercise and environmental stress) or unknown breed-specific differences in the effects of dystrophin deficiency [48]. Production of revertant fibres is thought to contribute to the phenotypic variation seen in humans and animals; revertant fibres are thought to be synthesised by rare, isolated, somatic reversions that re-establish the open reading frame and translation re-initiation [49, 50]. However, the alternative splicing with associated revertant fibres would not be possible in these MPs due to the nature of the mutation i.e. the complete loss of the entire *DMD* gene. Uncovering the responsible factors for phenotypic variation in DD-MD may uncover additional factors influencing disease progression and severity, and reveal further therapeutic targets.

Dystrophin is also found in the brain [9] where aberrant expression leads to mental retardation in approximately one third of affected human patients [51], and memory deficits, autism, attention deficit hyperactivity disorder and behavioural problems have also been reported [52]. To the authors’ knowledge, cognitive deficiency has not been reported in any DMD canine model to date. The lethargy, learning difficulties and episodes of abnormal behaviour observed in dogs 1 and 2 and episodes of decreased awareness in dog 2 may have resulted from a lack of dystrophin in the brain. No other cause for these cognitive and behavioural abnormalities was identified following extensive diagnostic investigations.

A major limitation of the present study is the incomplete picture of clinical signs and disease progression, caused by three factors. The first is the low number (three) of dystrophin-deficient MPs examined. Second, the clinical examinations of the three affected dogs were based on their clinical signs and as a result some tests were not conducted in all three dogs. Finally, the dogs were not followed closely throughout their lifetimes, and one was lost to follow-up at a young age, resulting in a less complete picture of clinical signs and disease progression. All dogs examined were privately owned pet dogs examined and diagnosed at the request of their owners, and not members of a research colony. We would argue that the welfare of the dogs was the primary focus throughout, and our findings are opportunistic but still contribute to muscular dystrophy research in humans and animals, whilst adhering to the Three Rs principle.

The causal mutation for DD-MD has been identified in many breeds of dogs [15–22]. The mutation types are varied, and include simple exonic substitutions, frameshifting insertions and deletions and splice site mutations, amongst others. We now report that a family of dystrophic MPs has a deletion mutation spanning >5.6 Mb and encompassing the entirety of the *DMD* gene, a mutation similar to the only other natural whole-*DMD* gene deletion described in an animal model, in the GSHP [17, 36]. The precise deletion breakpoints could not be determined in either the MP or the GSHP, as the breakpoint regions (which overlap in the two breeds), are highly homologous to one another and other regions of the X chromosome (Fig 5).

Dogs with DD-MD are valuable models for disease research and for evaluation of treatments for DMD and BMD in humans. These MPs represent good animal models for dystrophin gene therapy trials and/or myoblast transfer. Given the lack of *DMD* gene in these individuals, any *DMD* transcripts or dystrophin detected in their tissues after therapeutic trial, could only be produced by the dystrophin delivery vehicle, simplifying the interpretation of experimental results. Furthermore, although systemic genetic therapy is showing promise in the treatment of DMD, there is some concern for the immunogenicity of dystrophin, as this protein is not recognised as own in DMD patients, who have never synthesised normal dystrophin. Given the lack of *DMD* gene, *DMD* transcripts and dystrophin, these MPs, together with the GSHP model, should prove useful for genetic therapies, providing the most sensitive prediction of immune responses to these therapies [36].

X chromosome deletions that encompass the *DMD* gene and adjacent genes could result in additional clinical signs to those caused by aberrant or deficient dystrophin. Indeed, combinations of DMD, retinitis pigmentosa, chronic granulomatous disease, adrenal gland insufficiency, glycerol kinase deficiency and McLeod syndrome have been reported in human medicine [53–55]. The deletion described in the MP-DD-MD model is at least 5.6 Mb in size (X:26,239,786–31,867,079) encompassing the entire *DMD* gene together with 23 adjacent genes and three pseudogenes (S3 Table). Of the 23 genes, 12 were RNA genes and 11 were protein-coding genes (S3 Table). None of the protein-encoding genes have been reported to have functions that could be associated with any of the clinical signs displayed by these MPs, and most of the clinical signs observed in the affected MPs, if not all, may be attributed to dystrophin deficiency. Nevertheless, it remains possible that the loss of one or more of the genes within the deletion, apart from *DMD*, resulted in clinical effects that we did not detect at the time of examination, or with the investigation methods used.

The precise deletion breakpoints could not be identified in the MP-DD-MD model, for several possible reasons. Firstly, part of the genome near the 5’ end of the deletion remains unannotated in CanFam3.1 (711 bp; X:26,238,732–26,239,442) and whole genome sequencing data suggests that the breakpoint may be within this region (Fig 5). Secondly, regions of the genome in which the sequence remains unknown are often, in our experience, very difficult to interrogate; they tend to be GC-rich, presumably resulting in increased secondary structure. Analysis of the nucleotide sequence in this region, determined by VanBelzen et al. [36], reveals a high GC content of 75%, supporting this theory. Thirdly, much of the known sequence in the 5’- and 3’-breakpoint regions is homologous to many genomic regions. As a result, we were unable to design primers specific to only the targeted regions and were unable to define the deletion breakpoints. Consequently, we were unable to design a conventional assay to identify female carriers of the deletion. We therefore developed a relative qPCR-type assay, using primers and a probe that targeted sequences within the deleted region, and reference primers and a probe that targeted sequences elsewhere on the X chromosome. However, this assay is not specific to this MP *DMD* deletion, and could therefore result in a false positive in the unlikely occurrence of a different deletion encompassing the probe.

The UK Kennel Club only registers MPs with solid coat colours, while parti coloured MPs usually have solid-coloured patches over a white coat. Harlequin is a subtype of parti coat colouring with a defined saddle area and points (as found in a Dobermann). As DD-MD has not been reported in any other miniature, toy or standard poodle, and the deletion was absent from a larger cohort of 44 MPs, it is likely that the deletion described here is either confined to this Harlequin-coloured MP family or is exceedingly rare.

In summary, we have described a family of MP dogs in which DD-MD segregates in a sex-linked, recessive manner. Furthermore, we identified a large deletion encompassing the entire *DMD* gene, which was only seen in this family. To the authors’ knowledge, this deletion, together with that of the GSHP DD-MD model, is the largest mutation affecting the *DMD* gene identified so far, including the human *DMD* gene, and thus expands the genetic variability associated with muscular dystrophy in dog breeds and humans.

## Supporting information

S1 Table Primers, PCR conditions and expected and observed PCR products. (XLSX)

S2 Table Summary of the clinical and diagnostic findings, treatment and outcome of dogs 1–4. (XLSX)

S3 Table Genes and proteins within the region deleted in Harlequin MP. (XLSX)

## References

[1] Koenig M, Monaco AP, Kunkel LM. The complete sequence of dystrophin predicts a rod-shaped cytoskeletal protein. Cell. 1988;53(2):219–28. . 3282674 10.1016/0092-8674(88)90383-2

[2] Mendell JR, Shilling C, Leslie ND, Flanigan KM, al-Dahhak R, Gastier-Foster J, et al Evidence-based path to newborn screening for Duchenne muscular dystrophy. Annals of neurology. 2012;71(3):304–13. doi: 10.1002/ana.23528 . 22451200 10.1002/ana.23528

[3] Caskey CT, Nussbaum RL, Cohan LC, Pollack L. Sporadic occurrence of Duchenne muscular dystrophy: evidence for new mutation. Clin Genet. 1980;18(5):329–41. . 7460369 10.1111/j.1399-0004.1980.tb02293.x

[4] Nowak KJ, Davies KE. Duchenne muscular dystrophy and dystrophin: pathogenesis and opportunities for treatment. EMBO Rep. 2004;5(9):872–6. doi: 10.1038/sj.embor.7400221 ; PubMed Central PMCID: PMCPMC1299132. 15470384 10.1038/sj.embor.7400221 PMC1299132

[5] Hoffman EP, Brown RH Jr., Kunkel LM. Dystrophin: the protein product of the Duchenne muscular dystrophy locus. Cell. 1987;51(6):919–28. . 3319190 10.1016/0092-8674(87)90579-4

[6] Aartsma-Rus A, Van Deutekom JC, Fokkema IF, Van Ommen GJ, Den Dunnen JT. Entries in the Leiden Duchenne muscular dystrophy mutation database: an overview of mutation types and paradoxical cases that confirm the reading-frame rule. Muscle Nerve 2006;34:135–44. doi: 10.1002/mus.20586 16770791 10.1002/mus.20586

[7] Monaco AP, Bertelson CJ, Liechti-Gallati S, Moser H, Kunkel LM. An explanation for the phenotypic differences between patients bearing partial deletions of the DMD locus. Genomics. 1988;2(1):90–5. . 3384440 10.1016/0888-7543(88)90113-9

[8] Collins CA, Morgan JE. Duchenne’s muscular dystrophy: animal models used to investigate pathogenesis and develop therapeutic strategies. Int J Exp Pathol. 2003;84:165–72. doi: 10.1046/j.1365-2613.2003.00354.x 14632630 10.1046/j.1365-2613.2003.00354.x PMC2517561

[9] Shelton GD, Liu LA, Guo LT, Smith GK, Christiansen JS, Thomas WB, et al Muscular dystrophy in female dogs. J Vet Intern Med. 2001;15(3):240–4. . 11380033 10.1892/0891-6640(2001)015<0240:mdifd>2.3.co;2

[10] Goldstein JA, McNally EM. Perspectives on: SGP Symposium on Muscle in Health and Disease: mechanisms of muscle weakness in muscular dystrophy. The Journal of General Physiology. 2010;136:29–34. doi: 10.1085/jgp.201010436 20584890 10.1085/jgp.201010436 PMC2894554

[11] Bushby K, Bourke J, Bullock R, Eagle M, Gibson M, Quinby J. The multidisciplinary management of Duchenne muscular dystrophy. Current Paediatrics. 2005;15:292–300.

[12] Pichavant C, Aartsma-Rus A, Clemens PR, Davies KE, Dickson G, Takeda S, et al Current status of pharmaceutical and genetic therapeutic approaches to treat DMD. Mol Ther. 2011;19(5):830–40. doi: 10.1038/mt.2011.59 ; PubMed Central PMCID: PMCPMC3098643. 21468001 10.1038/mt.2011.59 PMC3098643

[13] Le Guiner C, Servais L, Montus M, Larcher T, Fraysse B, Moullec S, et al Long-term microdystrophin gene therapy is effective in a canine model of Duchenne muscular dystrophy. Nat Commun. 2017;25(8). 10.1038/ncomms16105 PMC5537486 28742067

[14] Shelton GD, Engvall E. Canine and feline models of human inherited muscle diseases. Neuromuscul Disord. 2005;15(2):127–38. doi: 10.1016/j.nmd.2004.10.019 . 15694134 10.1016/j.nmd.2004.10.019

[15] Sharp NJ, Kornegay JN, Van Camp SD, Herbstreith MH, Secore SL, Kettle S, et al An error in dystrophin mRNA processing in golden retriever muscular dystrophy, an animal homologue of Duchenne muscular dystrophy. Genomics. 1992;13(1):115–21. . 1577476 10.1016/0888-7543(92)90210-j

[16] Winand N, Pradham D, Cooper B. Molecular characterization of severe Duchene-type muscular dystrophy in a family of Rottweiler dogs Molecular Mechanisms of Neuromuscular Disease Muscular Dystrophy Association; Tucson, Ariz, USA 1994.

[17] Schatzberg SJ, Olby NJ, Breen M, Anderson LV, Langford CF, Dickens HF, et al Molecular analysis of a spontaneous dystrophin 'knockout' dog. Neuromuscul Disord. 1999;9(5):289–95. . 10407848 10.1016/s0960-8966(99)00011-5

[18] Walmsley GL, Arechavala-Gomeza V, Fernandez-Fuente M, Burke MM, Nagel N, Holder A, et al A duchenne muscular dystrophy gene hot spot mutation in dystrophin-deficient cavalier king charles spaniels is amenable to exon 51 skipping. PLoS One. 2010;5(1):e8647 doi: 10.1371/journal.pone.0008647 ; PubMed Central PMCID: PMCPMC2800183. 20072625 10.1371/journal.pone.0008647 PMC2800183

[19] Nghiem PP, Bello L, Balog-Alvarez C, Lopez SM, Bettis A, Barnett H, et al Whole genome sequencing reveals a 7 base-pair deletion in DMD exon 42 in a dog with muscular dystrophy. Mamm Genome. 2017;28(3–4):106–13. doi: 10.1007/s00335-016-9675-2 ; PubMed Central PMCID: PMCPMC5371640. 28028563 10.1007/s00335-016-9675-2 PMC5371640

[20] Smith BF, Yue Y, Woods PR, Kornegay JN, Shin JH, Williams RR, et al An intronic LINE-1 element insertion in the dystrophin gene aborts dystrophin expression and results in Duchenne-like muscular dystrophy in the corgi breed. Lab Invest. 2011;91(2):216–31. Epub 2010/08/18. doi: 10.1038/labinvest.2010.146 ; PubMed Central PMCID: PMCPMC2999660. 20714321 10.1038/labinvest.2010.146 PMC2999660

[21] Jenkins CA, Forman OP. Identification of a novel frameshift mutation in the DMD gene as the cause of muscular dystrophy in a Norfolk terrier dog. Canine Genet Epidemiol. 2015;2:7 doi: 10.1186/s40575-015-0019-4 ; PubMed Central PMCID: PMCPMC4579383. 26401335 10.1186/s40575-015-0019-4 PMC4579383

[22] Atencia-Fernandez S, Shiel RE, Mooney CT, Nolan CM. Muscular dystrophy in the Japanese Spitz: an inversion disrupts the DMD and RPGR genes. Anim Genet. 2015;46(2):175–84. doi: 10.1111/age.12266 . 25644216 10.1111/age.12266

[23] Bulfield G, Siller WG, Wight PA, Moore KJ. X chromosome-linked muscular dystrophy (mdx) in the mouse. Proc Natl Acad Sci U S A. 1984;81(4):1189–92. Epub 1984/02/01. ; PubMed Central PMCID: PMCPMC344791. 6583703 10.1073/pnas.81.4.1189 PMC344791

[24] Carpenter JL, Hoffman EP, Romanul FC, Kunkel LM, Rosales RK, Ma NS, et al Feline muscular dystrophy with dystrophin deficiency. Am J Pathol. 1989;135(5):909–19. Epub 1989/11/01. ; PubMed Central PMCID: PMCPMC1880103. 2683799 PMC1880103

[25] Klymiuk N, Blutke A, Graf A, Krause S, Burkhardt K, Wuensch A, et al Dystrophin-deficient pigs provide new insights into the hierarchy of physiological derangements of dystrophic muscle. Human molecular genetics. 2013;22(21):4368–82. Epub 2013/06/21. doi: 10.1093/hmg/ddt287 . 23784375 10.1093/hmg/ddt287

[26] Nakamura K, Fujii W, Tsuboi M, Tanihata J, Teramoto N, Takeuchi S, et al Generation of muscular dystrophy model rats with a CRISPR/Cas system. Sci Rep. 2014;4:5635 Epub 2014/07/10. doi: 10.1038/srep05635 ; PubMed Central PMCID: PMCPMC4088098. 25005781 10.1038/srep05635 PMC4088098

[27] Larcher T, Lafoux A, Tesson L, Remy S, Thepenier V, Francois V, et al Characterization of dystrophin deficient rats: a new model for Duchenne muscular dystrophy. PLoS One. 2014;9(10):e110371 Epub 2014/10/14. doi: 10.1371/journal.pone.0110371 ; PubMed Central PMCID: PMCPMC4195719. 25310701 10.1371/journal.pone.0110371 PMC4195719

[28] Kornegay JN, Bogan JR, Bogan DJ, Childers MK, Li J, Nghiem P, et al Canine models of Duchenne muscular dystrophy and their use in therapeutic strategies. Mamm Genome. 2012;23:85–108. doi: 10.1007/s00335-011-9382-y 22218699 10.1007/s00335-011-9382-y PMC3911884

[29] McGreevy JW, Hakim CH, McIntosh MA, Duan D. Animal models of Duchenne muscular dystrophy: from basic mechanisms to gene therapy. Dis Model Mech. 2015;8(3):195–213. doi: 10.1242/dmm.018424 ; PubMed Central PMCID: PMCPMC4348559. 25740330 10.1242/dmm.018424 PMC4348559

[30] Dubowitz V, Sewry CA, Lane RJM, Oldfors A, Company WBS, Elsevier. Histological and histochemical stains and reactions Muscle Biopsy: A Practical Approach. Philadelphia: Saunders/Elsevier; 2013 p. 16–27.

[31] Guo LT, Moore SA, Forcales S, Engvall E, Shelton GD. Evaluation of commercial dysferlin antibodies on canine, mouse and human skeletal muscle. Neuromuscul Disord. 2010;20(12):820–5. doi: 10.1016/j.nmd.2010.07.278 . 20817457 10.1016/j.nmd.2010.07.278

[32] Li H, Durbin R. Fast and accurate short read alignment with Burrows-Wheeler transform. Bioinformatics. 2009;25(14):1754–60. doi: 10.1093/bioinformatics/btp324 ; PubMed Central PMCID: PMCPMC2705234. 19451168 10.1093/bioinformatics/btp324 PMC2705234

[33] McKenna A, Hanna M, Banks E, Sivachenko A, Cibulskis K, Kernytsky A, et al The Genome Analysis Toolkit: a MapReduce framework for analyzing next-generation DNA sequencing data. Genome Res. 2010;20(9):1297–303. doi: 10.1101/gr.107524.110 ; PubMed Central PMCID: PMCPMC2928508. 20644199 10.1101/gr.107524.110 PMC2928508

[34] Rozen S, Skaletsky H. Primer3 on the WWW for general users and for biologist programmers. Methods Mol Biol. 2000;132:365–86. doi: 10.1385/1-59259-192-2:365 . 10547847 10.1385/1-59259-192-2:365

[35] Livak KJ, Schmittgen TD. Analysis of relative gene expression data using real-time quantitative PCR and the 2(-Delta Delta C(T)) Method. Methods. 2001;25(4):402–8. doi: 10.1006/meth.2001.1262 . 11846609 10.1006/meth.2001.1262

[36] VanBelzen DJ, Malik AS, Henthorn PS, Kornegay JN, Stedman HH. Mechanism of Deletion Removing All Dystrophin Exons in a Canine Model for DMD Implicates Concerted Evolution of X Chromosome Pseudogenes. Mol Ther Methods Clin Dev. 2017;4:62–71. doi: 10.1016/j.omtm.2016.12.001 ; PubMed Central PMCID: PMCPMC5363321. 28344992 10.1016/j.omtm.2016.12.001 PMC5363321

[37] Cooper BJ, Winand NJ, Stedman H, Valentine BA, Hoffman EP, Kunkel LM, et al The homologue of the Duchenne locus is defective in X-linked muscular dystrophy of dogs. Nature. 1988;334:154–56. doi: 10.1038/334154a0 3290691 10.1038/334154a0

[38] Hoogerwaard EM, Bakker E, Ippel PF, Oosterwijk JC, Majoor-Krakauer DF, Leschot NJ, et al Signs and symptoms of Duchenne muscular dystrophy and Becker muscular dystrophy among carriers in the Netherlands: a cohort study. Lancet. 1999;353:2116–19. 10382696 10.1016/s0140-6736(98)10028-4

[39] Hoogerwaard EM, van der Wouw PA, Wilde AAM, Bakker E, Ippel PF, Oosterwijk JC, et al Cardiac involvement in carriers of Duchenne and Becker muscular dystrophy. 1999;9:347–51. 10.1016/s0960-8966(99)00018-8 10407858

[40] Valentine BA, Winand NJ, Pradhan D, Moise NS, de Lahunta A, Kornegay JN, et al Canine X-linked muscular dystrophy as an animal model of Duchenne muscular dystrophy: a review. Am J Med Genet. 1992;42(3):352–6. doi: 10.1002/ajmg.1320420320 . 1536178 10.1002/ajmg.1320420320

[41] Shelton GD, Engvall E. Muscular dystrophies and other inherited myopathies. The Veterinary clinics of North America Small animal practice. 2002;32(1):103–24. . 11785725 10.1016/s0195-5616(03)00081-0

[42] Kornegay JN, Cundiff DD, Bogan DJ, Bogan JR, Okamura CS. The cranial sartorius muscle undergoes true hypertrophy in dogs with golden retriever muscular dystrophy. Neuromuscul Disord. 2003;13(6):493–500. . 12899877 10.1016/s0960-8966(03)00025-7

[43] Wetterman CA, Harkin KR, Cash WC, Nietfield JC, Shelton GD. Hypertrophic muscular dystrophy in a young dog. J Am Vet Med Assoc. 2000;16:878–81. 10.2460/javma.2000.216.878 22570900

[44] Baltzer WI, Calise DV, Levine JM, Shelton GD, Edwards JF, Steiner JM. Dystrophin-deficient muscular dystrophy in a Weimaraner. J Am Anim Hosp Assoc. 2007;43(4):227–32. doi: 10.5326/0430227 . 17615404 10.5326/0430227

[45] Pouwels S, de Boer A, Leufkens HG, Weber WE, Cooper C, van Onzenoort HA, et al Risk of fracture in patients with muscular dystrophies. Osteoporos Int. 2014;25(2):509–18. doi: 10.1007/s00198-013-2442-2 . 23948807 10.1007/s00198-013-2442-2

[46] Anil G, Guglielmi G, Peh WC. Radiology of osteoporosis. Radiol Clin North Am. 2010;48(3):497–518. doi: 10.1016/j.rcl.2010.02.016 . 20609888 10.1016/j.rcl.2010.02.016

[47] Olby NJ, Sharp NJ, Nghiem PE, Keene BW, DeFrancesco TC, Sidley JA, et al Clinical progression of X-linked muscular dystrophy in two German Shorthaired Pointers. J Am Vet Med Assoc. 2011;238(2):207–12. doi: 10.2460/javma.238.2.207 . 21235374 10.2460/javma.238.2.207

[48] Shelton GD. Three distinguishable phenotypes in Golden Retriever muscular dystrophy. Neuromuscular Disorders. 2009;19:800–1.

[49] Schatzberg SJ, Anderson LV, Wilton SD, Kornegay JN, Mann CJ, Solomon GG, et al Alternative dystrophin gene transcripts in golden retriever muscular dystrophy. Muscle Nerve. 1998;21(8):991–8. . 9655116 10.1002/(sici)1097-4598(199808)21:8<991::aid-mus2>3.0.co;2-0

[50] Winnard AV, Mendell JR, Prior TW, Florence J, Burghes AH. Frameshift deletions of exons 3–7 and revertant fibers in Duchenne muscular dystrophy: mechanisms of dystrophin production. Am J Hum Genet. 1995;56(1):158–66. ; PubMed Central PMCID: PMCPMC1801338. 7825572 PMC1801338

[51] Daoud F, Angeard N, Demerre B, Martie I, Benyaou R, Leturcq F, et al Analysis of Dp71 contribution in the severity of mental retardation through comparison of Duchenne and Becker patients differing by mutation consequences on Dp71 expression. Human Molecular Genetics. 2009;18 (20):3779–94. doi: 10.1093/hmg/ddp320 19602481 10.1093/hmg/ddp320

[52] Ricotti V, Mandy WP, Scoto M, Pane M, Deconinck N, Messina S, et al Neurodevelopmental, emotional, and behavioural problems in Duchenne muscular dystrophy in relation to underlying dystrophin gene mutations. Dev Med Child Neurol. 2016;58(1):77–84. doi: 10.1111/dmcn.12922 . 26365034 10.1111/dmcn.12922

[53] Francke U, Ochs HD, de Martinville B, Giacalone J, Lindgren V, Disteche C, et al Minor Xp21 chromosome deletion in a male associated with expression of Duchenne muscular dystrophy, chronic granulomatous disease, retinitis pigmentosa, and McLeod syndrome. Am J Hum Genet. 1985;37(2):250–67. ; PubMed Central PMCID: PMCPMC1684578. 4039107 PMC1684578

[54] Cole DE, Clarke LA, Riddell DC, Samson KA, Seltzer WK, Salisbury S. Congenital adrenal hypoplasia, Duchenne muscular dystrophy, and glycerol kinase deficiency: importance of laboratory investigations in delineating a contiguous gene deletion syndrome. Clin Chem. 1994;40(11 Pt 1):2099–103. . 7955386

[55] El Nemer W, Colin Y, Collec E, Gane P, Cartron JP, Kim CL. Analysis of deletions in three McLeod patients: exclusion of the XS locus from the Xp21.1-Xp21.2 region. Eur J Immunogenet. 2000;27(1):29–33. . 10651848 10.1046/j.1365-2370.2000.00188.x

